# The effect of biofeedback on smoking cessation—a systematic short review

**DOI:** 10.1007/s00508-021-01977-x

**Published:** 2021-12-06

**Authors:** Mohammad Keilani, Margarete Steiner, Richard Crevenna

**Affiliations:** Department of Physical Medicine, Rehabilitation and Occupational Medicine, Medical University of Vienna, Währinger Gürtel 18–20, 1090 Vienna, Austria

**Keywords:** Smoking, Cessation, Craving, Biofeedback, Neurofeedback

## Abstract

**Purpose:**

The aim of this systematic review was to focus on the effect of biofeedback on smoking cessation.

**Material and methods:**

This review was conducted following the PRISMA guidelines. Peer-reviewed original articles including biofeedback and/or neurofeedback training as an intervention for smoking cessation were included. The PubMed, MEDLINE, Web of Science, Scopus, and Cochrane Library databases were screened for trials published up to July 2021. The effects on smoking rates and smoking behavior, and biofeedback/neurofeedback training measures are summarized here.

**Results:**

In total, three articles fulfilled the inclusion criteria. The total Downs and Black checklist scores ranged from 11 to 23 points, showing that the articles were of poor to good methodological quality. The included studies were heterogeneous, both in terms of treatment protocols and in terms of outcome parameters. Pooling of data for a meta-analysis was not possible. Therefore, we were limited to describing the included studies. The included biofeedback study demonstrated that skin temperature training might improve the patients’ ability to raise their skin temperature aiming at stress alleviation. All three studies reported positive effects of biofeedback/neurofeedback in supporting smokers to quit. Furthermore, individualized electroencephalography neurofeedback training showed promising results in one study in modulating craving-related responses.

**Conclusion:**

The results of the present review suggest that biofeedback/neurofeedback training might facilitate smoking cessation by changing behavioral outcomes. Although the investigated studies contained heterogeneous methodologies, they showed interesting approaches that could be further investigated and elaborated. To improve the scientific evidence, prospective randomized controlled trials are needed to investigate biofeedback/neurofeedback in clinical settings for smoking cessation.

## Introduction

Smoking has an impact on almost all organs of the body and causes numerous diseases. It also affects the health of smokers in general and increases their mortality risk [1]. Smoking leads to nicotine dependence and so-called smoking habits, which are difficult to treat.

Smoking cessation significantly reduces the risk of smoking-related diseases and can add years of life. In Austria, 24% of men and 18% of women smoke daily [2]. Smoking cessation is essential for primary as well as tertiary prevention of several diseases, especially for cardiovascular and oncological diseases [3]. Smoking cessation notably lowers the risk of most health problems that result from smoking, including cancer, heart and lung diseases, as well as many other chronic health conditions. Furthermore, quitting smoking is an important part of the rehabilitation of smokers, and it prolongs the survival of smokers [3, 4].

There are several pharmacological as well as nonpharmacological treatment options to support smoking cessation as well as established concepts. [5]. These consist of combinations of first-line and second-line pharmacological interventions with advice and specialized counseling, including therapeutic education as well as behavioral treatment and are considered the best way for smokers to quit smoking [5, 6].

A major challenge for smoking cessation programs is relapse prevention. Relapse is triggered among others by psychosocial stress, as smoking is often used as a means of stress coping. Thus, ex-smokers are in need of alternative coping strategies [7–9]. Nonpharmacological approaches, such as biofeedback and neurofeedback can facilitate the self-regulation of predisposing relapse factors, such as craving and stress. While biofeedback is an active training with the aim of making different vegetative functions visible through recording respective body parameters (e.g. skin temperature, electrodermal activity, muscle tension), neurofeedback targets on the self-regulation of brain activity [8–10].

The aim of this short systematic review was to focus on the effect of treatment for smoking cessation through biofeedback.

## Methods

### Identification and selection of studies

A systematic review was conducted based on the Preferred Reporting Items for Systematic Reviews and Meta-Analysis (PRISMA) guidelines [11].

The PubMed, MEDLINE, Web of Science, Scopus, and Cochrane Library databases were screened for trials published from inception to July 2021. The search procedure included the terms “smoking”, “cessation”, “biofeedback”, and “neurofeedback” and their possible combinations. No filters were used. No restrictions were placed on the year of publication. Data were tabulated and a narrative synthesis was carried out, since the data heterogeneity did not allow for a meta-analysis. The modified Downs and Black checklist [12] was applied to assess the risk of bias.

### Inclusion and exclusion criteria

Any quantitative study type of primary and peer-reviewed research that included biofeedback and/or neurofeedback training as an intervention for smoking cessation was considered for inclusion. The inclusion criteria were as follows:

- Participants: current smokers, smoking more than 10 tobacco cigarettes per day
- Intervention: biofeedback and/or neurofeedback
- Control groups: no training, sham or other training, non-smokers, no control group
- Outcomes: effects on smoking rates, smoking behavior, biofeedback/neurofeedback training measures
- Study design: prospective, controlled and uncontrolled studies
- Language limitations: published in English or German

Retrospective trials, case reports, reviews, letters, editorials, commentaries, and conference papers were excluded.

### Quality assessment

Each included study was assessed for quality, using the modified version of the Downs and Black checklist [12]. This checklist includes 27 criteria, covering areas such as reporting quality, external and internal validity, and power. As previously suggested by Hooper et al. (2008) [13], item 27 (power) was modified compared to the original version (sample sizes have been calculated to detect a difference of ×% and y% with yes = 1 and no = 0). This modified checklist allows a maximum of 28 points. Score ranges were given corresponding to quality levels: excellent (26–28); good (20–25); fair (15–19); and poor (≤ 14). The quality of each study was independently assessed by two reviewers, with discrepancies resolved through discussion and consensus.

## Results

### Study selection

**Fig. 1:**
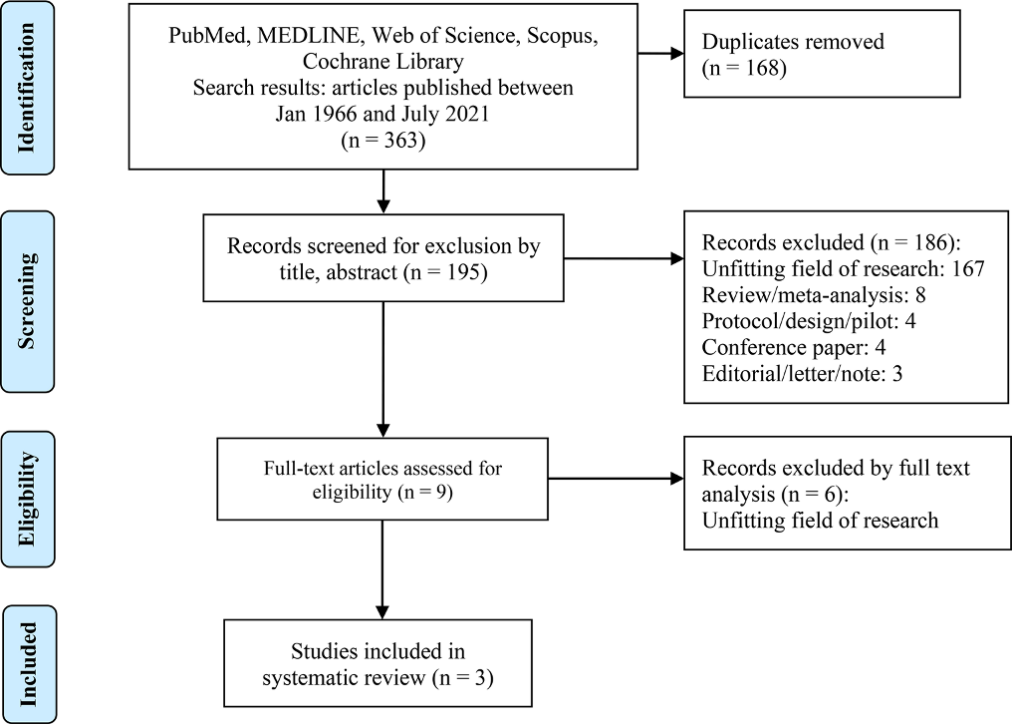
Flowchart of systematic literature search and selection according to PRISMA guidelines

A total of 363 relevant studies were identified. After the elimination of duplicates, 195 studies remained and were screened for eligibility based on the title and abstract. In total, 186 of these studies had to be excluded because they did not meet the inclusion criteria and 9 studies were selected for full-text analysis. Finally, three studies were included in the present review (Fig. 1).

### Risk of bias

The risk of bias analysis is summarized in Table 1. The total scores for the modified Downs and Black checklist [12, 13] ranged from 11 to 23 points (Table 1). A weakness of all three studies was that there was no comprehensive attempt made to measure adverse events (Table 1, item 8). Furthermore, none of the three studies reported the proportion of the source population from which the patients were derived (Table 1, item 11) and none of the studies presented main confounders (Table 1, item 25). A further weakness of two of the three included studies was that the study participants could not be blinded to the interventions (Table 1, item 14). In two of the studies, there was no attempt made to blind those measuring the main outcomes of the interventions (Table 1, item 15). Two of the three included trials were non-randomized studies (Table 1, item 23). With respect to the question of “was the randomized assignment concealed from both patients and health care staff until recruitment was complete and irrevocable”, two studies did not fulfil this criterion (Table 1, item 24). In one study, the characteristics of the included patients were not clearly described (Table 1, item 3). Furthermore, a list of principal confounders was not provided in one study (Table 1, item 5). In one study, the proportion of those asked to participate who thereafter agreed was not stated (Table 1, item 12). The loss of patients to follow-up was not taken into account in one study (Table 1, item 26). Furthermore, in three studies one item was not fulfilled: estimates of the random variability in the data for the main outcomes (Table 1, item 7), characteristics of patients lost to follow-up (Table 1, item 9), report of actual probability values (Table 1, item 10), information concerning the source of patients included in the study (Table 1, item 21), and the time period over which patients were recruited (Table 1, item 22).

**Table 1:** Methodological quality assessment: risk of bias evaluation of the included studies using the modified Downs and Black checklist [12, 13]

Study	Downs and Black checklist items																											Results	
	Reporting										External validity			Internal validity—bias							Internal validity—confounding (selection bias)						Power		
	1	2	3	4	5	6	7	8	9	10	11	12	13	14	15	16	17	18	19	20	21	22	23	24	25	26	27	Total	Quality level
Pandria et al. 2018 [14]	1	1	1	1	2	1	1	0	1	1	0	1	1	0	0	1	1	1	1	1	1	1	0	0	0	0	0	21	Good
Bu et al. 2019 [15]	1	1	1	1	1	1	1	0	1	1	0	1	1	1	1	1	1	1	1	1	1	1	1	1	0	1	0	23	Good
Griffith et al. 1983 [16]	1	1	0	1	0	1	0	0	0	0	0	0	1	0	0	1	1	1	1	1	0	0	0	0	0	1	0	11	Poor

### Intervention

#### Biofeedback studies

One non-controlled biofeedback study [14] could be included. The characteristics of this study are presented in Table 2.

**Table 2:** Study characteristics and results

Study	Study sample	Study design Controls/comparators	Intervention/assessment	Results
Pandria et al. 2018 [14]	*N* = 27 (mean age: 50.52 ± 12.364 a, males:females = 9:18)	Pretest-posttest	BF: 5 skin temperature training sessions of 30 min.	↓ Fagerström Test in males (*p* = 0.024)
	Median cig./d: 20 (r: 12.5–60)	No controls	Assessment: Baseline evaluation (session 1 and 2) and posttraining evaluation (sessions 3 and 4): clinical, behavioral, and electrophysiological evaluation	↓ General Health Test in males (*p* = 0.031)
	Mean smoking dependence: 358.5 ± 152.95 months		Questionnaires: Fagerström Test for Nicotine Dependence, Motivation, Contemplation Ladder, Minnesota Nicotine Withdrawal Scale, Beck Depression Inventory, State-Trait Anxiety Inventory, General Health Test, Rosenberg Self-Esteem Scale, World Health Organization Quality of Life-Brief	No change of CO levels after BF (*p* = 0.6)
				↓ Number of participants with moderate nicotine dependence decreased from 11 to 10
				↓ Number of participants with high nicotine dependence decreased 11 to 8
				EEG: Slight ↑ outflow of left vlPFC (*p* = 0.065), slight ↑ outflow of right TPC (*p* = 0.059)
				Females: ↑ outflow of right TPC (*p* = 0.028)
				Males: left mPFC (*p* = 0.003) and left PCU (*p* = 0.005)
				Reduced daily cigarettes: not reported
				Ratio of smokers who quit: not reported
Bu et al. 2019 [15]	*N* = 60 (age: 18–40a, males)	RCT	NFB: 2 training sessions of 1 h, interval: 1–2 days	*Effects on event-related potential correlates of cigarette craving*:
	Cig./d: > 10 for 2 years or more	Real-NFB group (*n* = 28) vs. yoked-NFB group (*n* = 25): brain activity pattern of matched participants	No smoking 2 h prior to training sessions	Real-NFB group: ↓ in craving-related P300 amplitudes compared with the yoked-NFB group (group-time interaction effect: *p* = 0.028)
			Visit 1: Baseline evaluation: clinical and behavioral assessment (TCQ, craving-related P300)	*Short-term effects on cigarette craving*:
			Visit 2 + 3: 2 NFB training sessions: smoking cue reactivity task (smoking-related images and neutral images)	Craving (TCQ): real-NFB group ↓ craving score from pre-NFB to post-NFB (*p* = 0.00021)
			Visit 4: Post-training behavioral session	*Long-term effects on smoking behavior*:
			Visit 5: Follow-up session	Real-NFB group: ↓ cig./day compared to the yoked-NFG
			Assessment: P300 pre-NFB and post-NFB	↓ cig./d at follow-up: 1 week (30.6%), 1 month (38.2%), 4 months (27.4%)
			TCQ: Cig. craving at pre-NFB (before 1. training session) and post-NFB (after 2. training session)	Yoked-NFB group: ↓ cig./d at follow-up: 1 week (14%), 1 month (13.7%), 4 months (5.9%)
			Interview: cig./day	Group differences were significant at 1 week (*p* = 0.01), 1 month (*p* < 0.005), 4 month (*p* = 0.03)
			Cig./d at baseline, 1 week, 1 month, 4 months after final neurofeedback visit	Ratio of smokers who quit: not reported
Griffith and Crossman 1983 [16]	*N* = 6 (age: not reported, males)	Pretest-Posttest	*NFB, 8 daily 30* *min sessions*:	During smoking a cig.: ↑ 4–8 Hz activity (*n* = 4)
	Moderate or heavy smokers (16–24 or > 35 cig./d)	No controls	No smoking 1 h prior toNFB	↓ 8–12 Hz activity (*n* = 6), ↑ HR (*n* = 5)
			*Phases*:	Immediately after smoking a cig.: no consistent brain wave change, ↑ HR (*n* = 5), ↓ skin temperature (*n* = 4)
			A1: Baseline	NFB: ↑ time spent producing 8–12 Hz compared to baseline levels (*n* = 4)
			B: Smoking a cigarette	Fadeout: participants (*n* = 2) able to produce 8–12 Hz activity levels for higher percentage of time without audible feedback at the end of the fadeout phase had quitted smoking at 6‑month follow-up, the other participants reduced daily cigarettes to 12–61% (*n* = 4)
			A2: Baseline	
			C: NFB: learn to produce occipital 8–12 Hz (alpha waves) in EEG, music feedback, eyes open	
			D: Fadeout: training to produce 8–12 Hz without audible feedback over 8 sessions	
			*Assessment*:	
			Occipital EEG, HR, hand skin temperature, blood pressure, behavioral data (Smoker’s Self-Testing Kit, General Background Questionnaire, smoking frequency inside and outside the experimental setting)	

*BF* biofeedback, *min.* minutes, *cig./d* cigarette per day, *bpm* beats per minute, *↓* decrease, *↑* increase, *EEG* electroencephalography, *vlPFC* outflow of right ventrolateral prefrontal cortex, *TPC* temporal pole cortex, *mPFC* medial prefrontal cortex, *PCU* precuneus, *NFB* neurofeedback, *h* hour, *RCT* randomized controlled study, *TCQ* Tobacco Craving Questionnaire, *P300* Craving-related P300 component (300–550 ms), *Hz* hertz,* HR* heart rate

Pandria et al. (2018) [14] conducted a non-controlled study to examine the effect of skin temperature biofeedback on smoking status and possible neuroplastic effects. Clinical, behavioral, and neurophysiological resting-state electroencephalography (EEG) data were collected from 27 subjects before and after five 30-min sessions of skin temperature training. The assessment included behavioral tests and questionnaires. In addition, a spirometry test was performed. The results showed a significant improvement in the degree of nicotine dependence measured by the Fagerström test and the score of the General Health Questionnaire in males, but not in females. In addition, the number of participants with moderate and severe nicotine dependence decreased. In the EEG, an increase in the outflow of the right ventrolateral prefrontal cortex (vlPFC) and temporal pole cortex (TPC) was observed. Differences from pretraining to posttraining were obtained in both males and females (Table 2; [14]).

#### Neurofeedback studies

One randomized clinical trial and one non-controlled study were analyzed [15, 16]. The characteristics of these studies are presented in Table 2.

Bu et al. (2019) [15] conducted a double-blind randomized placebo-controlled study to investigate smoking cessation by the use of individualized neurofeedback. Short-term and long-term behavioral effects were evaluated and 60 male current smokers were included. The participants had consumed at least 10 cigarettes per day for at least 2 years. They received two neurofeedback training sessions (1 h/session), either from their own brain (real-neurofeedback group) or from the brain activity pattern of matched participants in the control group (yoked-neurofeedback group). Adaptive closed-loop training was used as an intervention to change EEG activity pattern for smoking cue reactivity. The craving-related P300 component (300–550 ms) of the event-related potential (ERP) was measured and evaluated at baseline and after neurofeedback. A multivariate pattern analysis of all EEG channel data corresponding to an evoked smoking cue reactivity task was performed. Cigarette craving and the number of cigarettes smoked per day were assessed. The procedure contained five stages. The results showed a notably decreased rate of cigarettes smoked per day in the real-neurofeedback group compared with the control group at the 1‑week, 1‑month, and 4‑month follow-ups. Furthermore, the craving score showed significant improvements for the real-neurofeedback group (*p* < 0.05), while the control group showed no significant changes. The real-neurofeedback group showed a significant decrease in cigarette craving and craving-related P300 amplitudes compared with the control group. Furthermore, a greater decrease in mean P300 amplitude within the real-neurofeedback group correlated with a greater decrease in craving score, but this was not the case in the control group. In addition, it was reported that the degree of deactivation during the first cycle of neurofeedback correlated significantly with the number of cigarettes smoked per day at the 4‑month follow-up for participants in the real-neurofeedback group (Table 2; [15]).

Griffith and Crossman (1983) [16] conducted a non-controlled pretest-posttest study to identify the physiological variables that can contribute to maintenance of cigarette smoking as well as to investigate whether smoking frequency decreases when individuals are trained via neurofeedback procedures with music feedback to increase the 8–12 Hz occipital EEG activity as a substitute for smoking (Table 2; [16]). They included six male moderate or heavy smokers (15–24 or over 35 cigarettes daily), selected based on motivation to quit smoking and alpha rhythm stability. The authors used multiple 30-min sessions in an eyes-open condition (baseline recordings, recordings during smoking, neurofeedback sessions, and fadeout sessions). The results revealed that while smoking a cigarette, all 6 smokers showed decreased 8–12 Hz occipital EEG activity and 4 of them increased 4–8 Hz activity. Immediately after smoking a cigarette, five smokers demonstrated a continual increase in their heart rates, four showed a decrease in skin temperature. No consistent specific EEG changes appeared within subjects immediately after they smoked a cigarette. During the neurofeedback training, four of six smokers increased the amount of time they were producing 8–12 Hz brain waves. Two out of the six participants, namely, those who retained the trained alpha modulation skill, had quit smoking at the end of the 6‑month follow-up period. The other four participants reduced daily cigarettes to 12–61% (Table 2; [16]).

## Discussion

The results of the present systematic review reveal that one randomized controlled study [15] and two pretest-posttest studies [14, 16] have aimed to evaluate biofeedback and/or neurofeedback training as an intervention for changing smoking behavior. One study reported the ratio of smokers who quit smoking [16]. Furthermore, two studies reported the number of cigarettes smoked per day after the intervention ([15, 16]; Table 2).

Pandria et al. [14] used hand finger temperature as a biofeedback training modality, with the goal for participants to obtain control over the functions of the autonomic nervous system. Positive effects of biofeedback on the ability to control skin temperature were reported. In this study, the severity of nicotine dependence was significantly reduced after biofeedback training in males. Furthermore, the number of participants with moderate and high nicotine dependence decreased (Table 2; [14]).

The included neurofeedback studies [15, 16] showed a positive effect of a neurofeedback intervention on the number of cigarettes smoked per day; however, both studies used different neurofeedback treatment protocols. Bu et al. (2019) used an individualized protocol in their study to train the EEG patterns associated with smoking cue reactivity, while Griffith and Crossman (1983) made use of occipital alpha (8–12 Hz) modulation protocols [15, 16]. Therefore, a comparison of the two studies is not possible (Table 2).

Biofeedback and neurofeedback are mental techniques that can be used to learn to control some of the body’s functions, such as measures of the autonomic nervous system [9]. Biofeedback and neurofeedback are interventions used within the framework of behavioral medicine and based on learning theory. The individual learns to change the biofeedback signal by controlling the body parameter. Positive reinforcement changes the anchor actions in memory and allows an increased degree of control over a previously involuntary function [9]. Stress is considered an aggravating factor promoting a “vicious cycle” of nicotine addiction, while stress-related conditions seem to be predisposing factors for relapse [8]. One of the major goals of the included studies was to support the self-regulation of predisposing relapse factors, especially craving and stress in smokers.

The results of the present review indicate that biofeedback and neurofeedback might be promising measures to support smokers to quit smoking. Nevertheless, the results of the present review should be interpreted with caution because there was heterogeneity of the treatment protocols as well as outcome parameters throughout the included studies. Pooling of data for meta-analysis was not possible. Therefore, we were limited to a narrative description of the included studies.

In comparison to biofeedback, biomarker feedback is also used for smoking cessation. Biomarkers refer to biological indices, such as carbon monoxide (CO) levels (expired or carboxyhemoglobin) or cotinine levels (serum, saliva, or urinary). In this respect, Clair et al. concluded in their meta-analysis of randomized controlled studies in biomedical assessment that there were no significantly increased cessation rates from feedback of risk exposure, consisting of feedback on CO measurements [17]. On the other hand, more recently Marler et al. [18] performed a single-arm cohort 12-week study using a breath sensor that measured CO in the exhaled breath of smokers. Their results showed a significant increase in motivation to quit, a reduction of cigarettes smoked per day, and favorable quitting attempt rates [18].

In summary, it should be underlined that the aim of this systematic review was to provide an overview of the currently existing literature on biofeedback for smoking cessation. There might be positive effects of biofeedback as a behavioral measure, which could be taken into account in comprehensive treatment concepts for smoking cessation, where the method of biofeedback could play a role as an additive tool within established treatment regimens in prevention and rehabilitation of cardiovascular diseases and especially regarding various effects of smoking on oncological diseases [9, 19]. A limitation of this review is that only three studies could be included and the quality of these studies only reached a poor or good level; however, in future investigations, it will be necessary to improve the methodological quality through minimizing the methodological bias (for example missing control group, low sampling rate, or no standardized clinical outcome). Furthermore, more high-quality studies are urgently needed to find the best biofeedback protocol for smoking cessation.

## Conclusion

The results of the present review indicate that biofeedback/neurofeedback training might facilitate smoking cessation through changing behavioral outcomes. Although the investigated studies contained heterogeneous methodology, they showed different promising approaches that could be further investigated and elaborated. To improve the scientific evidence, prospective randomized controlled trials are needed to investigate biofeedback/neurofeedback in clinical settings for smoking cessation.
